# Supplementary material for: Credibility of preprints: an interdisciplinary survey of researchers
Source: R Soc Open Sci. 2020 Oct 28;7(10):201520. doi: 10.1098/rsos.201520 (PMC7657885; doi:10.1098/rsos.201520)

Credibility of Preprints Services

Of the 18 preprint service characteristics participants were asked to rate, a majority of respondents rated 14 as increasing the credibility of a service (see Figure 1). Many of these characteristics apply to a major current preprint services (e.g. indexed by google scholar, free for submitters and readers, indicators of which content has been published in a peer-review journal). However, some characteristics are more inconsistent (e.g. open source software, ability to assign DOIs to preprints, preprint submission to a journal from the preprint platform).

Of the three characteristics that were not seen as increasing credibility by a majority of respondents, two were related to how withdrawls of preprints were handled. Neither was rated negatively by a majority of respondents; instead there were relatively equal proportions of participants who saw these items as negative, neutral, and positive. This could indicate that, unlike the other characteristics, there isn't a great deal of agreement about what is the ideal or normative behavior around how services handle preprint withdrawals, and so there may be room to set standards.

The only item that was rated as decreasing the credibility of a service by a majority of respondents was a preprint service allowing anonymous posting of preprints. We had seen some concern in the past in communities that traditionally use double blind peer review that preprints would undermine this process, since reviewers could easily figure out the authors of submissions by looking for the article on a preprint server. We had included the item about anonymous posting of preprints to determine if communities might be interested in this option, which would mitigate the ability of preprints to undermine double blind peer-review. Based on the survey results, respondents overwhelmingly viewed this as negative to neutral for service credibility. This could indicate fears about preprints identifying article submitters have past, or that anonymous postings of preprints are not seen as a viable option for dealing with this issue.

In terms of characteristics that increased the credibility of a service broadly and were used to judge individual preprints, information related to reproducibility was rated highly by a majority of researchers for both. Information about the peer-review status of preprints was rated extremely highly for services overall but just shy of a majority rated this above the midpoint for individual preprints. This difference could be driven by the fact that we asked about publication for the service overall, while asking about *submission* to a peer-review journal for individual preprints. We saw the largest differences in questions related to commenting/endorsements. While comments and endorsements were rated as very or extremely important by a minority of respondents for the credibility of preprints, a majority stated that allowing endorsements of preprints would increase the credibility of a service. Given the fact that the questions were slightly different and used different scales, they can't be directly compared. But, it seems that researchers might like the *idea* of endorsement/commenting more than they actually use this information. A mismatch between valuing the ability to comment and using commenting corresponds well with current behavior on preprint services; many preprint services have commenting systems, but they appear to be very underused.

Figure 1

Response to the survey questions about how different characteristics affect the credibility of preprint services. For each question, the numbers to the left of the bars indicates the percentage of respondents who thought each characteristics would decrease the credibility of the service to some degree, the number in the center of the bar indicated the percentage who thought the characteristic wouldn't change the credibility of a service, and the number to the right of the bars indicates the percentage of respondents who thought each characteristic would increase the credibility of the service to some degree.

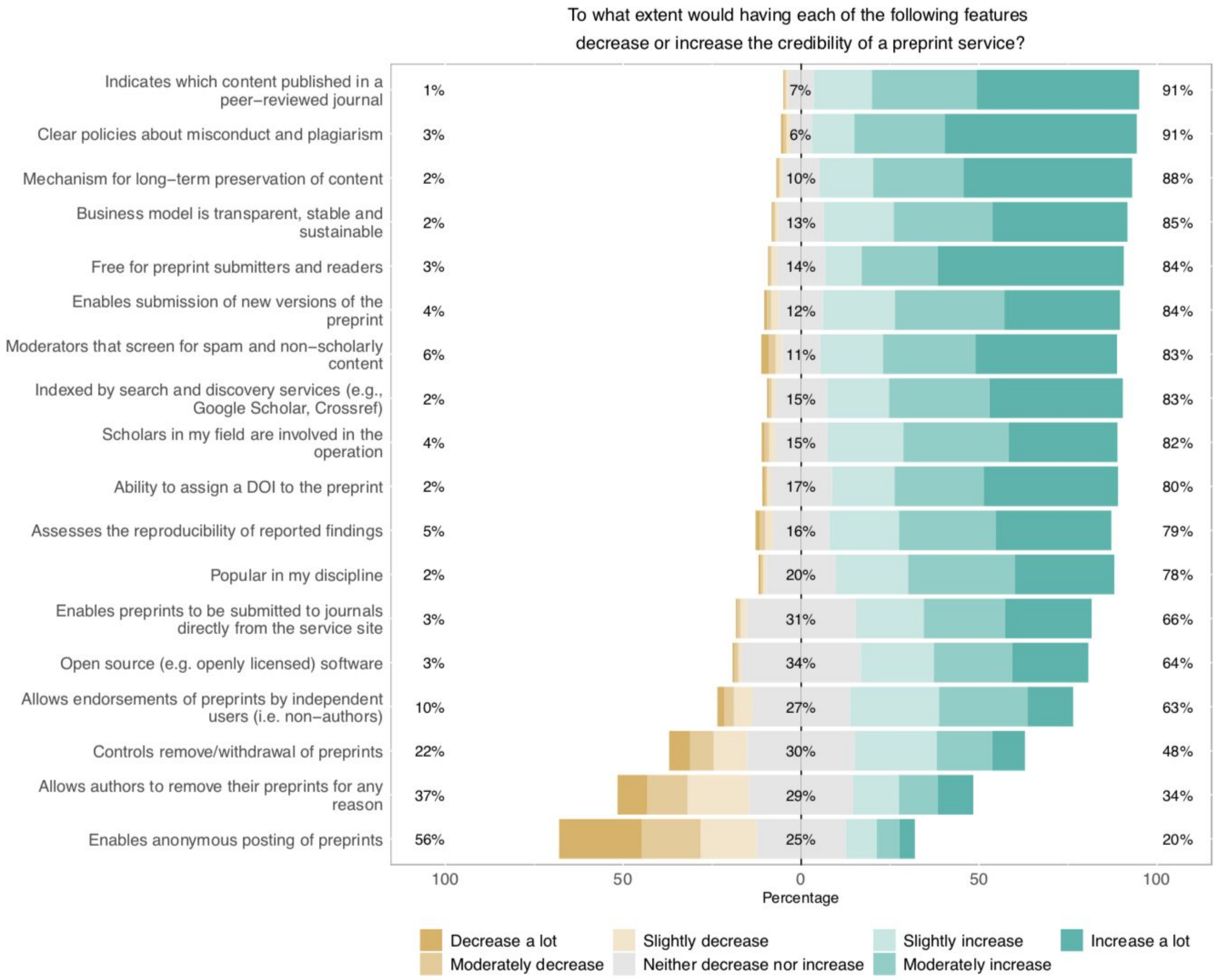

Supplement: Supplemental Analysis of Preprint Service Questions [file rsos201520supp1.pdf]
